# Procoagulant in vitro effects of clinical cellular therapeutics in a severely injured trauma population

**DOI:** 10.1002/sctm.19-0206

**Published:** 2020-01-06

**Authors:** Mitchell J. George, Karthik Prabhakara, Naama E. Toledano‐Furman, Brijesh S. Gill, Charles E. Wade, Bryan A. Cotton, Andrew P. Cap, Scott D. Olson, Charles S. Cox

**Affiliations:** ^1^ Department of Surgery McGovern Medical School at The University of Texas Health Science Center Houston Texas; ^2^ Department of Pediatric Surgery McGovern Medical School at The University of Texas Health Science Center Houston Texas; ^3^ U.S. Army Institute of Surgical Research JBSA‐FT Sam Houston San Antonio Texas

**Keywords:** adipose stem cells, adult hematopoietic stem cells, adult stem cells, bone marrow stromal cells, flow cytometry, heparin, mesenchymal stem cells, stem cells

## Abstract

Clinical trials in trauma populations are exploring the use of clinical cellular therapeutics (CCTs) like human mesenchymal stromal cells (MSC) and mononuclear cells (MNC). Recent studies demonstrate a procoagulant effect of these CCTs related to their expression of tissue factor (TF). We sought to examine this relationship in blood from severely injured trauma patients and identify methods to reverse this procoagulant effect. Human MSCs from bone marrow, adipose, and amniotic tissues and freshly isolated bone marrow MNC samples were tested. TF expression and phenotype were quantified using flow cytometry. CCTs were mixed individually with trauma patients' whole blood, assayed with thromboelastography (TEG), and compared with healthy subjects mixed with the same cell sources. Heparin was added to samples at increasing concentrations until TEG parameters normalized. Clotting time or R time in TEG decreased relative to the TF expression of the CCT treatment in a logarithmic fashion for trauma patients and healthy subjects. Nonlinear regression curves were significantly different with healthy subjects demonstrating greater relative decreases in TEG clotting time. In vitro coadministration of heparin normalized the procoagulant effect and required dose escalation based on TF expression. TF expression in human MSC and MNC has a procoagulant effect in blood from trauma patients and healthy subjects. The procoagulant effect is lower in trauma patients possibly because their clotting time is already accelerated. The procoagulant effect due to MSC/MNC TF expression could be useful in the bleeding trauma patient; however, it may emerge as a safety release criterion due to thrombotic risk. The TF procoagulant effect is reversible with heparin.

## Graphical abstract

Clinical cellular therapeutic (CCT) procoagulant effects in trauma and healthy subjects. Error bars represent a 95% confidence interval. CCTs have a greater procoagulant effect in healthy subjects from a tissue factor load of 250 to 1700. Trauma patients are hypercoaguable at baseline and have less physiological capacity for an additional procoagulant response.

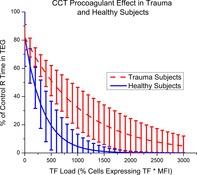

**Significance statement**

Stem cells are currently under investigation as a treatment for sequela of trauma like brain or lung injury. However, stem cells express tissue factor (TF) that causes rapid blood clotting. It is demonstrated that stem cells make blood from trauma patients, which clot faster. A potential antidote to this effect is heparin, a common and inexpensive blood thinner. It is believed that stem cells used in trauma studies should be risk‐stratified based on their TF expression.

## 1 INTRODUCTION

Traumatic injury is the leading cause of death in people ages 1 to 44 years old in the United States.^1^ Advances in resuscitation and hemorrhage control strategies have improved survival after injury.^2^ However, after initial stabilization, there are few treatments for the morbidity associated with major trauma. For example, therapy for sequela of traumatic brain injury (TBI) or hemorrhagic shock remains largely supportive.^3^, ^4^ Clinical cellular therapeutics (CCTs) like human mesenchymal stem cells (MSCs) and mononuclear cells (MNCs) may treat these deadly and morbid conditions.^5^, ^6^, ^7^ However, CCTs express tissue factor (TF) which is a potent activator of the coagulation cascade.

TF expression by CCTs has been linked with procoagulant effects in non‐trauma patients.^8^ In a previous broad survey of multiple tissue sources of CCTs from multiple donors, we demonstrated a causal relationship of CCT‐associated TF with accelerated clot formation using thromboelastography (TEG) and accelerated thrombin production using a calibrated thrombogram.^9^ Previous to that, Christy et al demonstrated similar findings in human adipose and bone marrow MSCs.^10^ In addition, Moll et al examined placental decidual and bone marrow stem cells, showing a relationship between TF expression and increased blood clotting.^11^, ^12^ However, the effects of TF expressed by MSCs or MNCs in a population of severely injured trauma patients are unknown.

The purpose of this study is to investigate the procoagulant activity of CCTs in a severely injured trauma population and identify methods of its reversal. We hypothesize that CCTs will have a procoagulant effect in trauma patients similar to healthy uninjured subjects and that this effect is reversible with standard anticoagulation drugs. We use TEG to measure the procoagulant effect of CCTs in trauma patients and add heparin to reverse this effect.

## 2 METHODS

### 2.1 Human subjects

This study was conducted at the Memorial Hermann Hospital within the Texas Medical Center, Houston, Texas. Prior to the study, approval was obtained from the Institutional Review Board (IRB) for blood collection from trauma subjects (HSC‐GEN‐12‐0059) and healthy controls (HSC‐MS‐10‐0190). Patients meeting the highest level of trauma team activation were included in the study from August 2017 to March 2018. Patients were excluded from the study if they were younger than 16 years, pregnant, prisoners, enrolled in other studies, found to be pharmacologically anticoagulated by antiplatelet agents such as aspirin or clopidogrel, or declined to give informed consent. The patients from whom we could not obtain an admission blood draw were also excluded from the study. Informed consent was obtained from the patient or a legally authorized representative within 72 hours of admission. A waiver of the informed consent was obtained for those patients who were discharged or died within 24 hours. In the remaining cases in which the informed consent could not be obtained, the patients were excluded from the study and their blood samples were destroyed.

### 2.2 Blood sample collection

Blood samples from trauma subjects were drawn with the initial sample within 5 minutes after arrival to the hospital. Two milliliters of blood were drawn through venipuncture into a Vacutainer tube containing 3.2% citrate and inverted to assure proper anticoagulation. Samples obtained from healthy subjects were collected in a similar manner. Vital signs, injury data, Injury Severity Score (ISS), laboratory data, and demographic variables were also obtained from trauma subjects.

### 2.3 CCT preparation

CCTs were sourced from four different human tissues including amniotic fluid derived MSCs, adipose‐derived MSCs (ADP MSCs), bone marrow‐derived MSCs, and bone marrow MNCs. All tissues were acquired either from commercial sources or under IRB‐approved protocols.

Processing of the amniotic fluid‐derived MSCs (AF MSCs) was carried out in an ISO Class 7 human cell production facility in compliance with current Good Manufacturing Practice (GMP) guidelines of the FDA. Amniotic fluid samples were collected through the approved IRB protocol HSC‐MS‐11‐0593. All reagents used were of GMP grade, and risk analysis of the manufacturing process was performed as previously described.^13^ In brief, amniotic fluid was centrifuged at 400*g* for 15 minutes and the pellet was resuspended in sterile‐filtered complete TheraPEAK XenoFree chemically defined mesenchymal stromal cell growth medium (Lonza, Walkersville, Maryland) supplemented with 20% allogeneic pooled human AB serum (Valley Biomedical, Winchester, Pennsylvania) and 5 ng/mL basic fibroblast growth factor (CellGenix, Freiburg, Germany). Cells were plated on Corning (Corning, New York) CellBIND surface and incubated at 37°C in a 5% CO_2_ and 95% relative humidity environment. Nonadherent cells were removed after 48 hours, and growth medium was changed every 3‐5 days. Upon reaching 70% confluence, cells were rinsed with calcium‐ and magnesium‐free phosphate‐buffered saline (PBS), detached with TrypLE Express XenoFree reagent (Thermo Fisher Scientific, Waltham, Massachusetts) and progressively passed and transferred to the scale‐appropriate cell‐culture platform for expansion. Cells were frozen in CryoStor CS10 (Biolife Solutions, Bothell, Washington) animal protein‐free, defined cryopreservation medium and stored in a liquid nitrogen vapor freezer.

Primary adipose biopsy samples were kindly provided by Dr. Saverio LaFrancesca and designated for research use only. ADP MSCs were isolated by washing the tissue three times in cold alpha‐MEM (Sigma Aldrich) containing 50 μg/mL gentamicin and mincing tissue into 5 mm pieces. The tissue was digested in a buffer containing alpha‐MEM, 300 IU/mL of Collagenase Type II (Worthington Biochemicals), 50 μg/mL gentamicin, and 1% bovine serum albumin 7.5% (Fraction, Gibco) for 55 minutes at 37°C/5% CO_2_. For every 3 g of the tissue, 10 mL of digestion buffer was used. After incubation, the tubes were centrifuged at 400*g* for 15 minutes at room temperature. The cell pellet was plated at a density of 9 g tissue/225 cm^2^ Flasks (Thermo). Cells were expanded in 5% Platelet Lysate (Gulf coast blood bank) in alpha‐MEM, 1000 U/mL heparin, and 10 μg/mL gentamicin. Passage 0 was maintained at 37°C/5% CO_2_, fed every third day until confluence reached 70%. Upon reaching the desired confluence, the medium was discarded, the cultures were washed with PBS, and the adherent cells harvested with 0.25% trypsin/1 mM EDTA for 5 minutes at 37°C and frozen at 10^6^ cells per milliliter in a cryosolution containing 10% dimethyl sulfoxide (DMSO; Cryostor CS10) for subsequent experiments.

Bone marrow‐derived MSCs (BM MSCs) were obtained from fresh bone marrow through the approved IRB protocol HSC‐MS‐08‐0393 and expanded following established procedures.^13^ Briefly, BM MSCs were cultured in complete culture medium that consisted of alpha‐minimal essential medium (Life Technologies, Grand Island, New York), 17% fetal bovine serum (FBS; lot‐selected for a rapid growth of MSC; Atlanta Biologicals, Norcross, Georgia), 100 units/mL penicillin (Thermo Fisher Scientific), 100 mg/mL streptomycin (Life Technologies), and 2 mM l‐glutamine (Thermo Fisher Scientific). BM MSCs were incubated with medium replaced every 2 days until 70% confluence. Medium was then discarded, cultures were washed with PBS, and adherent cells were harvested with 0.25% trypsin/1 mM EDTA (Thermo Fisher Scientific) for 5 minutes at 37°C and frozen at 10^6^ cells per milliliter for subsequent experiments.

Bone marrow mononuclear cells (BM MNCs) were isolated from fresh whole bone marrow from a commercial source (AllCells, Emeryville, California) according to common protocols using density centrifugation. Briefly, bone marrow from a healthy donor was diluted 1:2 with PBS and layered on top of Ficoll‐Paque (GE Healthcare) and centrifuged at 400*g* for 30 minutes at room temperature. The MNC layer was carefully collected and rinsed twice with PBS, and BM MNCs were suspended in RPMI media supplemented with 10% heat‐inactivated FBS (AtlantaBio, Atlanta, Georgia). Aliquots of 1 × 10^7^ cells/mL were cryopreserved by the addition of 10% DMSO, followed by freezing and storage in the vapor phase of a liquid nitrogen freezer for future use.

Cell preparation for testing occurred on weekday mornings at 8 am when at least one trauma patient had arrived overnight after 4 am For experiments described below, cells were thawed, counted using a hemocytometer, and resuspended in PBS to a working concentration of 10^6^ cells/mL. Cells were not used beyond 12 hours after preparation. There was a single donor for each type of cell, with 1 × 10^6^ cells aliquoted to individual vials to allow testing on multiple days.

### 2.4 Thromboelastography

TEG is a viscoelastic assay that measures coagulation kinetics of whole blood or platelet‐rich plasma. Primary metrics include R time that reflects time to initial fibrin formation, maximal amplitude (MA) that reflects the contribution of fibrin and platelets to ultimate clot strength, alpha angle that reflects the rate of fibrin formation, and lysis (LY30) that reflects the degree of fibrinolysis 30 minutes after the MA is reached.

TEG was performed using the Haemoscope TEG 5000 Coagulation Analyzer (Haemoscope Corp., Niles, Illinois). The final assay volume in the TEG cup was 360 μL which included 20 μL of 0.2 M CaCl_2_, 36 μL of either recombinant TF dilutions or CCTs suspended in PBS at a concentration 10^6^ cells/mL and 304 μL of citrated whole blood. CaCl_2_ was added to the TEG cup first followed by recombinant TF dilutions or CCTs and blood was added last. The sample was mixed twice using a pipette before starting the assay. The final molarity of CaCl_2_ was 0.01 M. The final concentration of CCTs was 10^5^ cells/mL, which is similar to clinical trials administering CCTs which use a concentration of 1 to 10 × 10^6^ cells/kg.^7^, ^10^ Control assays had an identical volume of PBS vehicle added to each sample. Each sample was run in duplicate, and values for TEG parameters were averaged. Each trauma patient and control sample were assayed with the four different CCTs and compared to their controls. TEG was performed within 6 hours of collection for trauma patients and within 2 hours of collection for healthy controls. These TEG data are separate from the rapid TEG performed in the hospital, presented in Table 2.

### 2.5 Flow cytometry

Cells were evaluated for the percent cells expressing mesenchymal markers CD29, CD44, CD73, CD90, CD105, CD31, CD34, CD45, and HLA‐DR using multiparametric flow cytometry panels (BD Biosciences). Mouse anti‐human CD142 (BD Biosciences) was included in the panel to determine the level of TF expression on the cell surface. Cells were washed and suspended in staining buffer (Biolegend) at a concentration of 0.5 × 10^6^/100 μL. Antibodies were added, followed by a 20‐minute incubation at room temperature. 7AAD was added to exclude dead cells, and the solution was diluted to a total volume of 1 mL. Flow analysis was performed on a Gallios (Beckman Coulter) and analyzed using the Kaluza v. 1.5a analysis software (Beckman Coulter). Results presented are percent of expression relative to controls of unstained cells, isotype controls, and fluorescence minus one controls (Table 1). Flow cytometry was performed on CCT samples at 9 am on weekday mornings after their preparation.

**Table 1:** Mesenchymal markers of AF MSC, ADP MSC, BM MSC, UMB MSC, and BM MNC

	7AAD	CD105	CD73	CD34	CD45	CD90	HLA DR	CD44	CD29	CD31	CD142
AF MSC	88	++	+++	−	−	+++	−	+++	+++	−	+++
ADP MSC	99	++	+++	−	−	+++	−	+++	+++	−	+++
BM MSC	86	+++	+++	−	−	+++	−	+++	+++	−	++
BM MNC	87	−	−	−	−	+	−	+++	++	++	+

*Note:* “+++” indicates the marker is highly expressed, “++” moderately expressed, “+” expressed, and “−” indicates not detected.

Abbreviations: APN, average passage number; AF MSC, amniotic fluid‐derived mesenchymal stromal cell; ADP MSC, adipose‐derived MSCs; BM MNC, bone marrow‐derived mononuclear cell; BM MSC, bone marrow‐derived MSC; UMB MSC, umbilical cord MSC.

### 2.6 Calculation of TF load

TF load in CCT samples was measured in a manner described previously.^9^ Briefly, the product of the mean fluorescent intensity (MFI) and percent cells expressing the anti‐human CD142 antibody, or TF, from flow cytometry were calculated as relative units. MFI reflects the density of antibody detected on a single cell. The MFI is calculated from the arithmetic mean for all cells expressing TF. Expressing CCT TF in this manner reflects both the percentage of cells expressing TF in a sample and their average density of expressed TF on the cell surface.

### 2.7 Reversal of the procoagulant effect with heparin

Increasing concentrations of heparin were added to whole blood treated with CCTs and tested as described above in TEG. The heparin dose was increased for each CCT treatment until TEG R time returned to normal. Heparinization of blood samples was done with different heparin concentrations to achieve samples with a final concentration of 0.1, 0.2, 0.5, 1, 2, and 4 U/mL. Control samples were prepared in similar ratios with vehicle only.

### 2.8 Data Analysis

Correlation between TF load and TEG for both trauma and healthy subjects was determined with Pearson's product moment correlations. Statistical significance was set to *P* < .05. Nonlinear regression curves were fitted to TF load and TEG data for trauma and control subjects. Based on variance of the source data, 95% confidence intervals were calculated for each curve at TF steps of 250. Analysis was performed using Stata 14.2 (StataCorp LLC, College Station, Texas) and graphically presented using Origin 8 (OriginLab Corp., Northampton, Massachusetts).

## 3 RESULTS

### 3.1 Trauma patient characteristics, laboratory values, and baseline coagulation function

Thirty‐six trauma patients were enrolled into this study. Patient demographics, vital signs, laboratory data, and coagulation function measured by rapid TEG are presented in Table 2. Mean age of the cohort was 41 ± 18 years, 73% were male, and ISS was 13 ± 10 with 8% mortality. Of note, 75% of patients had blunt injury patterns. Mean pH was 7.29, base excess was −3, and lactate was 3.8. Mean rapid TEG values performed in hospital were within normal limits.

**Table 2:** Patient demographics, injury, vitals, laboratory values, and coagulation function in all patients

	All patients (*N* = 36)	Reference ranges
**Demographic data**		
Age (years)	41 ± 18	
Male (%)	73	
**Injury and vitals**		
Blunt (%)	75	
Systolic BP (mm Hg)	137 ± 27	
Heart rate (beats per min)	109 ± 14	
Glasgow Coma Score (GCS)	13 ± 4	
Injury Severity Score (ISS): 0.5‐1	13 ± 10	
Mortality, % (N)	8.3 (3)	
**Laboratory values**		
pH	7.29 ± 0.12	7.35 to 7.45
Base excess (mEq/L)	−3.1 ± 5.6	−2.0 to 2.0
WBC (×10^9^/L)	12.2 ± 5.6	4.5 to 11
Lactate (mmol/L	3.8 ± 3.7	0.5 to 1
Platelet count (K/μL)	237 ± 73	150 to 450
**Coagulation function**		
TEG ACT (s)	109 ± 15	80 to 140
TEG angle	74 ± 4.6	66 to 82
TEG MA	65 ± 4.9	52 to 72
TEG LY30 (%)	1.8 ± 1.6	0 to 3

*Note:* Mean and SD values are reported.

Abbreviations: ACT, activated clotting time; MA, maximum amplitude; TEG, thromboelastography.

### 3.2 CCTs accelerate clot formation in trauma patients

**Figure 1:**
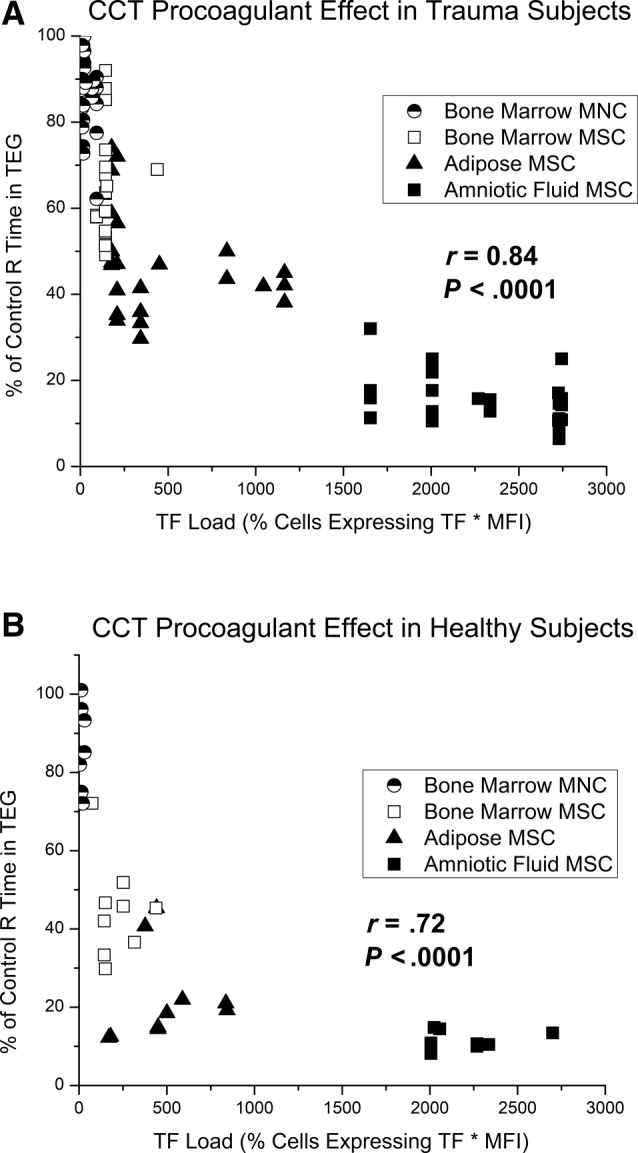
A, Pearson's correlation between relative TEG R time and tissue factor (TF) load in trauma patient blood samples. Each blood sample was treated with amniotic fluid MSC, adipose MSC, bone marrow MSC, or bone marrow MNC in separate TEG assays to determine a percent change in R time from controls. TF load was determined on the day of testing using flow cytometry. CCTs that express high levels of TF decrease R time more than those that express low levels of TF. Pearson' correlation of conglomerate data was excellent with *r* of .84, *P* < .0001. B, Pearson's correlation between relative TEG R time and TF load in healthy subject blood samples. CCT, clinical cellular therapeutic; MNC, mononuclear cell; MSC, mesenchymal stromal cell

Blood samples from the 36 trauma patients were assayed in standard TEG with the four different CCTs. TEG R time decreases as TF load increases with a Pearson's product‐moment correlation *r* value of .84 and *P* < .0001 (Figure 1A). For CCTs like BM MNC, TF load is low and R time is very near control values for each patient. AF MSC has a much higher TF load with resultant R times below 10% of control values.

### 3.3 CCT procoagulant effects in trauma patients vs healthy controls

**Figure 2:**
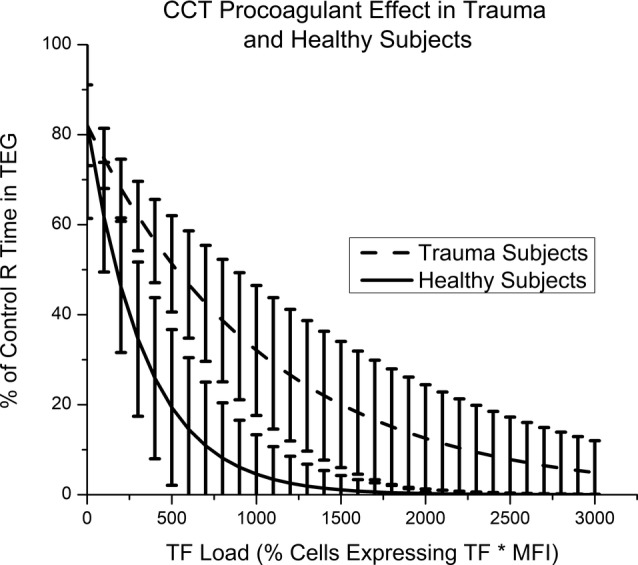
Clinical cellular therapeutic (CCT) procoagulant effects in trauma and healthy subjects. Error bars represent a 95% confidence interval. CCTs have a greater procoagulant effect in healthy subjects from a tissue factor (TF) load of 250 to 1700

Blood samples from 10 healthy subjects were also assayed in TEG with the four different CCTs. A Pearson's product‐moment correlation had an *r* value of .72 and *P* < .0001 (Figure 1B). Distribution of CCT TF was similar to those used in the trauma cohort, with AF MSC expressing the most TF and BM MNC expressing the least. Compared with trauma patients, healthy controls demonstrated greater relative decreases in TEG R time when treated with the same CCTs (Figure 2). Error bars represent 95% confidence intervals, and the cohorts demonstrate no overlap from TF load of 250 to 1700 relative units. Raw TEG data for trauma patients and healthy controls are presented in Table 3 as an average R time in minutes with and without each CCT treatment.

**Table 3:** Raw averaged values for TEG R time in healthy subjects and trauma patients, measured in minutes

	TEG R time (minutes)	
	Healthy subjects (N = 10)	Trauma patients (N = 36)
Control	8.0 ± 1.6	5.0 ± 2.7
Bone marrow MNC	6.1 ± 0.3	4.1 ± 1.2
Bone marrow MSC	3.5 ± 1.1	3.0 ± 0.8
Adipose MSC	1.7 ± 0.6	1.9 ± 0.7
Amniotic fluid MSC	1.3 ± 0.2	0.7 ± 0.2

*Note:* Controls and treatments with each CCT are presented for healthy subjects and trauma patients.

Abbreviations: MNC, mononuclear cell; MSC, mesenchymal stromal cell.

### 3.4 Heparin reverses the procoagulant effect of CCTs

**Figure 3:**
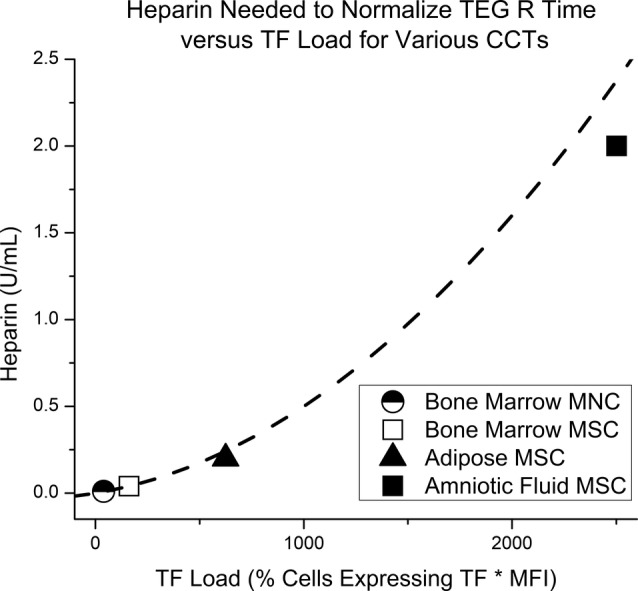
Heparin reverses the procoagulant effect of clinical cellular therapeutics (CCTs). As the tissue factor (TF) load of a given CCT increases, more heparin is needed to correct R time. Heparin required to correct R time versus TF load varies exponentially with an *r* value of .98

The procoagulant effect of CCTs is potentially reversible with standard anticoagulants. Varying concentrations of heparin were added to whole blood from a healthy control treated with the four different CCTs. For each CCT, TEG R time eventually returned to control values with increasing concentrations of heparin added. The amount of heparin in units per milliliter required to reverse the CCT procoagulant response in vitro increases as TF load increases (Figure 3). For each CCT, the concentration of heparin needed to correct R time can be converted to a single dose of heparin for an 80 kg patient. Appreciating the specific limitations of our in vitro test system, our data indicate that anticoagulation could be achieved at the following heparin doses: for the AF MSC sample 12 000 U is required, for the ADP MSC sample 1200 U is required, for the BM MSC sample 240 U is required, and for the BM MNC sample 60 U is required.

## 4 DISCUSSION

Our data demonstrate a procoagulant effect of CCTs in blood from severely injured trauma patients and healthy controls. TEG R time decreased in a cohort of 36 trauma patients as the TF load of the CCT treatment increased. The same CCTs had a stronger procoagulant effect in a cohort of healthy subjects. Heparin restored TEG R time to control values for each CCT tissue source. Doses of heparin required to restore R time increased as TF load of the CCT increased. This study is clinically relevant because CCTs are being introduced as novel therapy for TBI^7^ and acute respiratory distress syndrome.^14^ We show that CCTs accelerate clot formation in vitro in a cohort of trauma patients. In addition, we demonstrate that heparin can reverse this procoagulant effect. The procoagulant effects of CCTs in trauma patients must be considered as a safety release criterion for their clinical use. Consideration should be given to choosing cell sources expressing less TF and cell expansion methods that minimize TF expression should also be explored.^8^, ^15^

The present study in trauma patients is distinct from our previous study in a healthy subject. In our previous study, blood samples from a single healthy donor were treated in vitro with 33 different CCT donors from six different tissue sources.^9^ The focus of that study was to establish the causal relationship of CCT‐associated TF and acceleration of blood clotting. In the current study, blood samples from 36 trauma patients and 10 healthy controls were treated in vitro with four different CCTs. The focus of the present study was to demonstrate differences in the CCT procoagulant effect in trauma patients, which is important because they are a clinical population where CCTs have a potential benefit.

The primary finding in this study is an accelerated TEG R time of trauma subjects' whole blood due to treatment with TF‐expressing CCTs. Acceleration of TEG R time in trauma subjects correlates with deep vein thrombosis (DVT). Brill et al demonstrated that trauma patients with hypercoaguable TEG on admission have higher rates of DVT than those that do not (15.6% vs 8%, *P* = .039).^16^ Selby et al studied the coagulation markers thrombin, prothrombin, and soluble fibrin at different time points in trauma subjects. They found that soluble fibrin was increased in those who developed DVT compared with those who did not.^17^ This finding relates to TEG in that R time reflects initial formation of the fibrin clot.

Trauma patients are hypercoaguable after injury compared with uninjured subjects. A study by Differding et al compared TEG results in 46 severely injured trauma patients and 46 healthy controls. TEG R time was significantly faster in the trauma group (6.2 vs 8.9 minutes, *P* < .001).^18^ Schreiber et al demonstrated in another study that up to 65% of trauma patients are initially hypercoaguable based on the TEG R time, which drops to 25% by day 3 of admission.^19^ The hypercoaguable state of trauma subjects could explain the greater procoagulant effect of the CCTs in healthy subjects demonstrated in our study. Healthy subjects have a larger window for response to a CCT treatment because their R time is longer at baseline. Trauma subjects already have an accelerated rate of clot formation due to the natural reaction to trauma and have less physiological capacity for an additional procoagulant response. The clinical significance of this difference in procoagulant response to CCTs between healthy and trauma subjects is uncertain and clinically unimportant. The clinical relevance lies in that CCTs still have a significant procoagulant effect in trauma subjects.

Heparin is a safe, cheap, and effective anticoagulant commonly used clinically. The reversal mechanism of the CCT procoagulant effect by heparin is the inactivation of activated factor X, which limits the effects of TF activation of factor VII. We demonstrate that heparin can restore the TEG R time to normal values for the four different CCTs. AF MSCs express very high levels of TF and require more heparin than BM MNCs to restore R time to control values. For perspective, a common amount of heparin to bolus a patient for anticoagulation after a pulmonary embolus or a patient with unstable angina is 5000 U. To correct the procoagulant effect of AF MSCs in this study, we found that more than double that (12 000 U) was required to normalize the TEG R time. However, a recent study by Silachev et al potentially calls into question the efficacy of heparin treatment to ameliorate the procoagulant effects of CCTs.^20^ In their study, blood from newborn infants at high risk of thrombosis was collected 0.5‐1 hour after systemic heparinization at a dose of 5 U/h/kg. They demonstrated a persistent in vitro procoagulant effect of umbilical cord MSCs similar to non‐heparinized controls using rotational thromboelastometry rather than TEG. However, the dose of heparin used in the study was much lower than the standard 20‐30 U/h/kg dose for new born systemic heparinization.^21^ In addition, a bolus dose of heparin was not given, which could suggest that the ideal way to reverse the CCT procoagulant effect is with a bolus rather than a rate of heparin.

Although coadministration of heparin could be a solution to the procoagulant effect of CCTs, it raises a number of issues in the severely injured trauma patient. For example, heparin or other anticoagulants are contraindicated in patients with acute TBI due to high risk of worsening bleeding. Similarly, patients with solid organ injury such as liver, kidney, or spleen lacerations are placed at a higher risk for bleeding and prophylactic anticoagulation for venous thromboembolism is typically withheld for 24‐48 hours depending on the injury severity. Although in these cases where bleeding or hypocoagulability is an issue, the procoagulant effect of CCTs could be seen as an advantage. However, considering the procoagulant effect of CCTs as beneficial is unlikely since the lungs are a common site of microthrombi deposition in animal models.^22^

TF forms a perivascular envelope around blood vessels and is not normally present in blood.^23^, ^24^ Under normal conditions, TF is completely absent from circulating blood cells and in blood vessels only expressed in the adventitia.^25^, ^26^ TF introduced from injection of CCTs into the intravascular space causes an immediate coagulation reaction and leads to a consumption of coagulation factors. Moll et al named this procoagulant nature of CCT‐associated TF the instant blood‐mediated inflammatory reaction.^12^ Exogenous TF introduced in vivo by CCTs illicits an immediate consumption of coagulation factors and systemic formation of fibrin monomers. This induces a consumptive coagulopathy, and initial results from viscoelastic assays demonstrate faster onset of clotting.^8^ However, after this reaction is complete and the consumption coagulopathy is complete, TEG would likely demonstrate a slower onset of clotting, reduced rate of clot formation, and weaker clot strength. This potential discrepancy highlights the need for future studies examining the timing of the in vivo effect of CCT‐associated TF.

A limitation of this study was that CCTs are rarely given clinically in a single treatment, rather they are given in multiple doses over time. In this study we only modeled a single dose of CCTs.

## 5 CONCLUSIONS

Previous studies demonstrate an in vitro procoagulant response to CCTs related to their TF expression. These studies have been limited to blood donated by healthy subjects. Our study expands on previous work by confirming this effect in trauma patients. We demonstrate acceleration of clot formation in trauma patients as TF load of a CCT treatment increases. In addition, we demonstrate heparin as a potential reversal agent to this procoagulant effect.

## CONFLICT OF INTEREST

C.W. declared stock ownership with Decisio Health LLC that is unrelated to research and research funding from Grifols and Prytime that was paid to the institution. S.O. declared research funding from Hope Bio, Biostage, Athersys, and CBR. C.C. declared patient holder in Cellvation, Inc., consultant/advisory role with Cellvation Inc., Athersys Inc., Hope Biosciences, CBR, Inc., Biostage, Inc., stock ownership with Cellvation, Inc., Coagulex, Inc., Biostage, Inc., and research funding with Cellvation, Inc., Hope Bio. All the remaining authors declare no potential conflicts of interest.

## AUTHOR CONTRIBUTIONS

M.G.: conception and design, collection and analysis of data, manuscript writing; K.P., N.E.T.‐F.: collection of data, manuscript writing; B.S.G.: final approval of manuscript, conception and design, data analysis; C.E.W., B.A.C.: final approval of manuscript, manuscript writing, provision of patients; A.P.C.: final approval of manuscript, conception and design; S.D.O.: data analysis, manuscript writing, assembly of data; C.S.C.: final approval of manuscript, conception and design, data analysis, writing.

## Data Availability

The data that support the findings of this study are available from the corresponding author upon reasonable request.
